# Progress for Immunotherapy in Inflammatory Breast Cancer and Emerging Barriers to Therapeutic Efficacy

**DOI:** 10.3390/cancers13112543

**Published:** 2021-05-22

**Authors:** Regina M. Koch, Daniel R. Principe, Jose L. Cataneo, Ajay Rana

**Affiliations:** 1 Department of Medicine, University of Illinois at Chicago, Chicago, IL 60612, USA; rkoch3@uic.edu; 2 Department of Surgery, Division of Surgical Oncology, University of Illinois at Chicago, Chicago, IL 60612, USA; Principe@illinois.edu (D.R.P.); jcatan5@uic.edu (J.L.C.); 3 Medical Scientist Training Program, University of Illinois College of Medicine, Chicago, IL 60612, USA; 4 Jesse Brown VA Medical Center, Chicago, IL 60612, USA

**Keywords:** inflammatory breast cancer, immunotherapy, immune checkpoint inhibitors, tumor microenvironment, tumor immunology

## Abstract

**Simple Summary:**

Despite recent advances in the treatment of other breast cancer subtypes, inflammatory breast cancer (IBC) remains a significant clinical challenge, with an overall 5-year survival rate of 39%. Though immunotherapy has shown remarkable efficacy in other difficult-to-treat cancers, such approaches have yet to show substantial therapeutic efficacy in IBC. Here, we summarize the known immune composition of IBC tumors, as well as past and present efforts to advance immunotherapy in the treatment of IBC.

**Abstract:**

Inflammatory breast cancer (IBC) is a rare and aggressive subtype of breast cancer that carries a particularly poor prognosis. Despite the efficacy of immunotherapy in other difficult to treat forms of breast cancer, progress for immunotherapy in IBC has been difficult. Though immunotherapy has been under clinical investigation in IBC since the 1970s, few approaches have shown significant therapeutic efficacy, and no immunotherapy regimens are currently used in the treatment of IBC. Here, we provide a comprehensive summary of what is known about the immune composition of IBC tumors, clinical and basic science evidence describing the role for immune checkpoints such as PD-L1 in IBC pathobiology, as well as past and present attempts to advance ICIs in the treatment of IBC.

## 1. Introduction

While clinical outcomes are improving for most breast cancer subtypes, inflammatory breast cancer (IBC) still carries a poor prognosis with an overall 5-year survival rate of 39% [1]. IBC is a rare subtype of locally advanced, invasive breast carcinoma that typically presents with pain, erythema, and severe swelling of the affected breast [2]. These clinical features of IBC develop rapidly, which can lead to a misdiagnosis of cellulitis, mastitis, or another infectious process [2]. Given the severe swelling often associated with IBC, early reports proposed the disease stemmed from an acute inflammatory response to an underlying breast carcinoma. However, it has since been suggested that IBC is not necessarily rooted in inflammation, and the swelling and erythema associated with IBC are presumed to be due to the obstruction of dermal lymphatic vessels by tumor microemboli [3].

As IBC has reached the lymphatic vessels to cause these characteristic changes in the skin, it is considered to be at least a stage III breast cancer. Current treatment guidelines for stage III IBC recommend neoadjuvant chemotherapy followed by surgical excision and adjuvant radiation [4]. Currently, the National Comprehensive Cancer Network (NCCN) guidelines recommend that patients who have shown adequate response to neoadjuvant chemotherapy undergo total mastectomy with level I/II axillary lymph node dissection. This is recommended as soon as 3 weeks after completing systemic therapy in the absence of complications; however, timing can vary significantly based on the individual needs of the patient, and a longer interval is often preferred to allow for adequate recovery following neoadjuvant treatment [5,6]. For HER2-enriched tumors, pertuzumab and trastuzumab combination therapy is also indicated, as is hormonal therapy for HR-positive tumors [4]. For stage IV disease, patients are treated with chemotherapy and any appropriate targeted therapy described above, with surgery and radiation used on a more case-by-case basis [4]. While this approach has modestly improved clinical outcomes for IBC patients, the overall prognosis remains poor [7]. For instance, patients with stage III IBC have an approximate 43% increased risk of death compared to those with non-IBC breast cancers [8], with a similar increase in mortality for patients with stage IV IBC [9]. Accordingly, despite comprising less than 2% of all breast cancers in the United States, IBC accounts for roughly 7% of breast cancer-related deaths [10,11], highlighting an urgent and unmet need for new therapeutic approaches for IBC.

Several such approaches are emerging in clinical trials, though results have been mixed and several promising trials were discontinued due to issues of patient enrollment. Notable examples include the combination of trastuzumab, docetaxel, and carboplatin as first line therapy for HER2-positive stage II/III IBC, which was terminated due to slow accrual in 2013 (NCT00118053). The combination of paclitaxel with anti-HER2 agents trastuzumab and pertuzumab is currently under investigation as neoadjuvant therapy for HER2-positive IBC (NCT01796197), though results have not yet been posted.

The anti-VEGF antibody bevacizumab has also been explored in IBC. In a now completed trial, bevacizumab was evaluated in combination with doxorubicin and docetaxel (NCT00016549), though responses were varied in the 19 patients that have been reported and the data were inconclusive [12]. A phase II trial has also evaluated nab-paclitaxel with or without bevacizumab preceded or followed by doxorubicin, cyclophosphamide, and pegfilgrastim as neoadjuvant therapy, though results have not yet been published (NCT00856492). Recently, results were shared from a European phase II trial of bevacizumab with paclitaxel, carboplatin, and cyclophosphamide with or without trastuzumab and endocrine therapy in the neoadjuvant setting. This approach was associated with a high rate of pathologic complete responses, particularly for HER2-positive IBC, as well as improved overall and disease-free survival [13].

Select studies have also evaluated the relationship between bevacizumab and HER2 status. For example, the phase II BEVERLY-1 trial (NCT00820547) enrolled 100 patients with HER2-negative IBC, and evaluated the combination of neoadjuvant fluorouracil, epirubicin, cyclophosphamide, and bevacizumab during for four cycles, followed by docetaxel and bevacizumab for another for four cycles. Between 2 and 4 weeks after surgery, patients received adjuvant radiotherapy, hormone therapy if indicated, and adjuvant intravenous bevacizumab. Unfortunately, the results suggested that the addition of bevacizumab does not provide clinical benefit to patients with non-metastatic HER2-negative IBC [14]. The BEVERLY-2 trial (NCT00717405) evaluated this approach in 52 patients with HER2-positive IBC. These results appear to suggest that the addition of bevacizumab is efficacious and well tolerated in patients [15], though further study is required prior to advancing bevacizumab in the treatment of IBC [16].

However, despite these and other clinical trials, overall outcomes for IBC remain poor. Immune checkpoint inhibitors (ICIs) have transformed the treatment paradigm of several cancer histologies. These strategies consist of neutralizing antibodies against negative immune checkpoints as Cytotoxic T-Lymphocyte Associated Protein 4 (CTLA-4), Programmed Cell Death 1 (PD-1), and PD-1 Ligand-1 (PD-L1), thereby impeding the ability of cancer cells to evade the immune surveillance program [17]. ICIs have shown remarkable clinical efficacy in the management of melanoma, lung cancer, head and neck cancers, and many others [18], with emerging evidence in many other difficult to treat cancers, including those of the esophagus, liver, and pancreas [19,20,21,22,23,24]. However, despite the continued progress for ICIs in other forms of breast cancer [25], relatively little is known regarding the potential for ICIs in IBC. Here, we summarize emerging clinical and basic science evidence describing the potential role of various immune cell types and immune checkpoints such as PD-L1 in IBC pathobiology, as well as past and present attempts to advance ICIs in the treatment of IBC.

## 2. Immune Composition of the IBC Microenvironment

**Figure 1:**
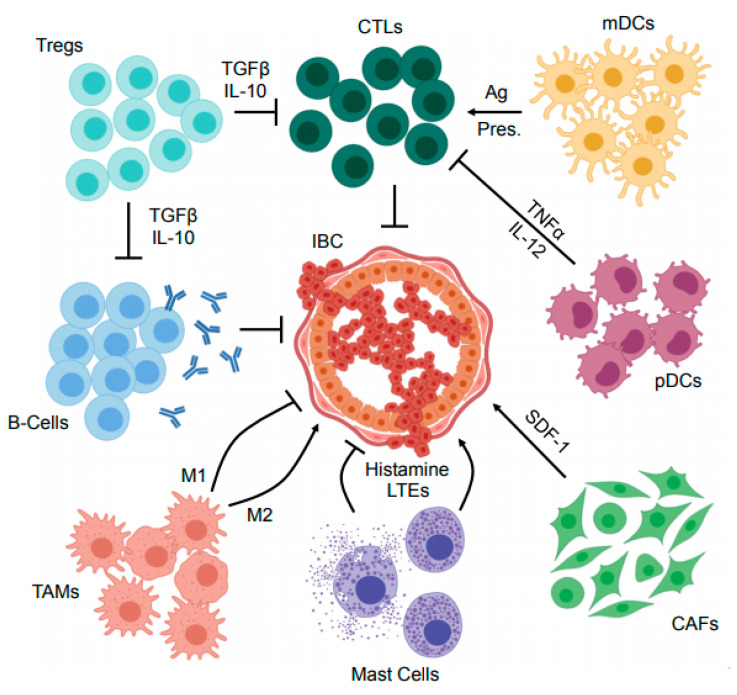
Mechanisms of immune suppression within the inflammatory breast cancer microenvironment. The inflammatory breast cancer (IBC) tumor microenvironment (TME) contains several immune and immunoregulatory cell types. Cytotoxic T-lymphocytes (CTLs) are central to the anti-tumor immune program, and restrain IBC development in cooperation with antigen (Ag) presenting cells such as myeloid dendritic cells (mDCs). Similarly, antibody producing B-cells have also been suggested to have a role in opposing IBC development. However, the IBC TME also harbors several immunosuppressive cell types such as regulatory T-cells (Tregs) that inhibit the effector function of anti-tumor T- and B-lymphocytes via several mechanisms including the secretion of suppressor cytokines such as transforming growth factor β (TGFβ) and IL-10. In addition to Tregs, several other cells have central roles in local immunosuppression including plasmacytoid dendritic cells (pDCs) that have been suggested to impair the action of CTLs via the secretion of cytokines including tumor necrosis factor α (TNFα) and IL-12. Mast cells have also been suggested to both enhance and oppose IBC progression through secretion of histamines and leukotrienes (LTEs), as have the heterogeneous population of tumor-associated macrophages (TAMs) that serve a variety of complex and seemingly paradoxical functions within the TME. Finally, cancer associated fibroblasts (CAFs) are emerging as an important immunomodulator in several breast cancers, and modulate a variety of cell processes via secretion of immunoregulatory proteins including stromal derived factor 1 (SDF-1). These and other cell types warrant further study in the complex and dynamic IBC TME, particularly in the setting of cancer immunotherapy.

There is mounting evidence suggesting that the tumor microenvironment (TME) is paramount to IBC pathobiology [26,27,28]. However, despite this well documented association, the composition of the IBC TME is very poorly described, particularly compared to that of other breast cancers. Histologically, IBC tumors have extensive involvement of lymphatics, blood vessels, cancer-associated fibroblasts and their associated extracellular matrix, as well as infiltrating immune cells [26,27,29,30], summarized in Figure 1. Here, we discuss the current consensus regarding the immune composition of the IBC TME, with a particular emphasis on those with known roles in IBC pathogenesis or prognosis.

## 3. Tumor Infiltrating Lymphocytes

Like other cancers, the immune infiltrate of the IBC TME appears to be heterogeneous. However, immune-related gene expression in IBC appears to be distinct from non-IBC tumors, independent of molecular subtype [31]. To date, most studies have focused on the resident lymphocytes of IBC tumors. For instance, it has been reported that up to 41% of IBC tumors harbor dense aggregates of CD8+ cytotoxic T-lymphocytes (CTLs) [32]. This was paralleled by a recent, larger study of 143 treatment naïve IBC patients, which reported that, on average, tumor-infiltrating lymphocytes (TILs) comprise roughly 18% of the tumor stroma [33]. The authors also found that tumors with a stroma comprised of over 10% leukocytes had a significant survival advantage [33]. This study also considered molecular subtype, examining hormone receptor and HER2 status. While they found no significant difference in TIL infiltration between IBC and non-IBC tumors in the luminal and triple-negative subgroups, HER2-enriched IBC tumors had increased TILs compared to HER2-enriched, non-IBC tumors [33].

Despite these observations, the role for TILs in IBC is poorly understood. A recent study has offered some insight into the potential contributions of TILs, in which the authors identified a 107-gene signature that predicted pathological complete response to neoadjuvant chemotherapy in IBC patients [34]. This gene signature included CD3, CD8, ITK, LCK, PIK3CD, and PTPN7, as well as several other genes involved CD8+ T cell activation and signaling [34]. Based on these observations, this group hypothesized that an adaptive T-cell response may predict responses to chemotherapy in IBC [34]. This was supported by a subsequent study that found that the increased presence of TILs is associated with improved responses to chemotherapy, conclusively implicating TIL density as the only significant positive prognostic factor beyond tumor staging at the time of diagnosis [33].

In addition to T-cell involvement, there are emerging data suggesting that B-cells also have important roles within the IBC TME. For instance, CD20+ B-cells are present in roughly 60% of IBC tumors [35]. Similar to previous findings concerning pan-TILs or CD8+ T-cells, patients with increased B-cell involvement had improved disease-free and overall survival, as well as an increased rate of pathologic complete response to neo-adjuvant chemotherapy [35]. Hence, while it is clear that both T and B-cells have prognostic relevance in IBC, the means through which they are recruited and how they restrain IBC progression are poorly understood and warrant further study.

## 4. Tumor Associated Macrophages

In addition to lymphocytes, the IBC TME also frequently harbors dense infiltrates of tumor-associated macrophages (TAMs) [36,37,38]. Interestingly, IBC tumors appear to generally have a more robust TAM infiltrate than non-IBC tumors [37]. The role of TAMs in other forms of breast cancer are only recently becoming clear, with evidence supporting a highly complex, two-way interaction between TAMs and tumor cells in breast cancer [39]. In IBC, tumor cells secrete various chemoattractants, including IL-8 and growth-regulated oncogene (GRO) chemokines, which recruit monocytes to the TME. There, they differentiate into tumor-promoting, immune-suppressing M2-like macrophages, secreting a variety of tumor-promoting factors such as IL-10 and VEGF, as well as additional IL-8 and GRO chemokines in a feed-forward loop [40]. Accordingly, TAM polarization has been shown to be a potentially useful prognostic factor in IBC, as an increased presence of IL-10 and VEGF expressing M2d macrophages has been suggested to promote tumor survival and progression as well as predict for early metastases and poor overall survival in other cancers [41,42,43].

Subsequent studies have explored the additional contributions of TAMs to the TME. For instance, cytokine array data suggest that TAMs from IBC tumors produce significantly more TNFα and IL-8 than TAMs from non-IBC breast cancers, both of which have established roles in tumorigenesis [37,44]. TAMs are also emerging as an important barrier to the effector function of cytotoxic T-lymphocytes, particularly in IBC. TAMs from IBC tumors produce several immunosuppressive cytokines, including CCL2 and IL-10 at higher levels than TAMs from non-IBC breast cancers [37]. Recently, the tetraspanin protein CD151 is emerging as a key mediator of monocyte/macrophage recruitment. IBC tumors with increased CD151 expression display increased TAM infiltration. This was recapitulated in patient-derived xenografts, where CD151 expression tumors displayed an increase in TAMs, mediated by increased expression of the monocyte chemoattractant midkine. The authors conclude that therapeutic inhibition of CD151/midkine signaling may offer a potential strategy to reshape the immune milieu of IBC tumors [45]. Hence, though the roles of TAMs in IBC are still emerging, it is clear that they serve an important role in shaping the IBC TME and warrant consideration when designing immune-based therapies.

## 5. Dendritic Cells

Dendritic cells (DCs) are emerging as an important and understudied component of the IBC immune milieu. Like TAMs, tumor-associated DCs are heterogeneous, consisting predominantly of myeloid DCs (mDCs) that mediate the adaptive anti-tumor response via activation of CD8+ T-cells and secretion of IL-12, IL-6, and TNF-alpha [46], and plasmacytoid DCs (pDCs) that mediate both adaptive and innate immune responses through antigen presentation and IFNα secretion [47]. In IBC, pDCs are more abundant in aggressive molecular/histological subtypes, and correlate with increased metastasis and poor overall survival [48,49]. Interestingly, other studies have suggested that these tumor-associated pDCs produce lower levels of IFNα when compared to peripheral blood pDCs, suggesting that they may remain refractory from full activation [50].

Though these data are unclear in IBC, other studies have explored pDC biology in other forms of breast cancer. While this is also still emerging, it appears that tumor-derived cytokines, including TGFβ and TNFα, impede pDC activation and IFNα biosynthesis [49]. These poorly active, IFNα-deficient pDCs appear to promote regulatory T-cell (Treg) expansion, thereby suppressing anti-tumor immune responses [49,50]. Though the role for pDCs is less established in IBC compared to other breast cancer subtypes, select studies suggest these findings may be applicable to IBC. This includes recent works highlighting the importance of IFNα in the IBC TME, as well as the association between functionally exhausted pDCs and the suppression of CD8-mediated cytotoxicity [51,52]. Hence, this area warrants continued exploration, particularly in the setting of IBC immunotherapy.

Recent evidence also suggests that mDCs may have divergent roles in IBC pathogenesis. Most notably, there is a clinical association between circulating tumor cells (CTCs) and peripheral mDC dysfunction. Specifically, IBC patients with increased CTCs had mDCs with impaired secretion of TNF-α before or after Toll-Like Receptor (TLR) stimulation, with similar results observed with IL-12. The authors suggested that this impaired secretion of TNF-α and IL-12 by peripheral mDCs may diminish T cell-mediated anti-tumor immunity, though this too warrants additional investigation [46].

## 6. Mast Cells

Though mast cells are often present in the TME of breast cancers, as with many other leukocyte subtypes described in this review, their role in IBC pathobiology and therapy remains unclear [53]. Clinically there is a positive association between mast cells and favorable prognosis in invasive, non-IBC breast cancer, thought to be mediated by anti-tumor cytolytic effects via mast cell secretion of histamine and leukotrienes [54]. However, these findings are contradicted by those from other groups suggesting that mast cell-derived factors enhance breast cancer angiogenesis and metastasis [55,56,57,58,59,60]. These results suggest that mast cells may be highly context-dependent, corroborated by additional studies reporting that mast cell activity may differ significantly in breast cancer depending on histological grade and/or molecular subtype [61,62].

Given this lack of consensus in more prevalent forms of breast cancer, it is unsurprising that the role of mast cells in IBC is also poorly understood. Early evidence suggests that mast cell infiltrates are present in many IBC tumors, and correlate with poor responses to neo-adjuvant chemotherapy independent of molecular subtype [63]. Given the spatial distribution of these mast cell infiltrates, the authors suggested that mast cells may suppress the effector function of adjacent immune cells and warrant further study as a novel immunosuppressant cell type within the IBC TME [63].

## 7. Cancer Associated Fibroblasts

Though classically associated with their role in the deposition of extracellular matrix proteins, cancer-associated fibroblasts (CAFs) are emerging as a central mediator of the neoplastic phenotype in several breast cancers, though they too are poorly established in IBC. Like many of the other cell types described previously, CAFs are better studied in other forms of breast cancer. For instance, CAFs have been shown to have driving roles in tumor initiation, promotion, and metastasis, as well as resistance to both chemo- and targeted therapies [64,65,66,67]. Accordingly, CAFs are now emerging as an important immunomodulation within the breast TME and an important consideration when designing immunotherapy regimens, though this has yet to be adapted to studies in IBC. In non-IBC tumors, CAFs have been demonstrated to recruit monocytes to the TME via secretion of stromal cell-derived factor-1 (SDF-1), and subsequently promote their polarization toward an immunosuppressive M2-like phenotype [68]. In non-IBC TNBC, CAFs also have the capacity to redirect resident M1-like macrophages to take on an M2-like phenotype. These CAF-educated TAMs have been shown to promote tumor cell proliferation/motility, as well as inhibit CD4+ T-cell proliferation [68].

This study also reported the presence of two phenotypically distinct myofibroblast subsets that differentially accumulate in TNBCs [68]. These subsets, deemed CAF-S1 and CAF-S4, respectively, were shown to have distinct properties and levels of activation, with the CAF-S1 cells having a clear role in mainlining the immunosuppressive breast TME. Specifically, tumors high in CAF-S1 cells were reported to have an increased accumulation of regulator T-cells, and were associated with poor prognosis. Similarly, these CAF-S1 cells enhanced CD4+ T-cell migration and subsequent differentiation into Tregs in vitro [68].

CAFs are also emerging as a potential candidate for therapy in other forms of breast cancer. For example, the use of a DNA vaccine targeting CAF activation had extensive ramifications on the breast TME in mice, reducing TAM and Treg accumulation and increasing CD8+ T-cell and DC accumulation. However, it is also important to note that treated tumors also displayed increased angiogenesis and lymphogenesis, again affirming the complex and multifaceted role of CAFs within the breast TME [69]. However, despite these and other important studies, there is relatively little known about the role of CAFs in IBC tumors, and the relevance of these findings to IBC pathobiology is only speculative. Hence, CAFs warrant continued exploration in IBC and may represent an important and understudied barrier to the efficacy of immune therapies.

## 8. Immune Checkpoints in IBC

Several studies have explored the expression of various immune checkpoints in IBC in hopes of advancing ICI-based treatments. Given the advent of PD-1/PD-L1 inhibitors in the treatment of other cancers, nearly all studies to date have focused on PD-L1 expression in IBC. A seminal study exploring PD-L1 status in IBC analyzed mRNA expression compared to both non-IBC breast tumors and normal breast tissue. When compared to normal breast tissue, 38% of patient IBC tumors had PD-L1 overexpression compared to 28% of non-IBC tumors [70]. Moreover, in IBC patients, overexpression of PD-L1 was associated with several clinical and molecular findings, including estrogen receptor-negative status, basal and ERBB2-enriched subtypes, and a gene signature suggestive of an apparent cytotoxic T-lymphocyte (CTL) response. Importantly, the authors found that PD-L1 overexpression was associated with improved responses to chemotherapy, independent of other variables [70].

Subsequent studies using immunohistochemistry have reported similar rates of PD-L1 expression in IBC. For instance, in a cohort of 143 treatment naïve IBC patients, 43% were positive for PD-L1 [33]. The authors also noted that PD-L1 expression was predominantly localized to the TILs, which were positive with an increased frequency than non-IBC breast tumors, independent of molecular subtype [33]. Another study including 221 treatment naïve IBC tumors similarly found that while 8% had PD-L1 expression on the tumor epithelium, 66% had PD-L1 expression in TILs [35]. Similarly, a study of 68 post-treatment IBC tumors found a PD-L1 positive rate of 37% [71]. Interestingly, the authors also found that PD-L1 expression was associated with reduced overall survival [71], contrasting other studies suggesting that PD-L1 overexpression was associated with improved responses to chemotherapy [70].

While the prognostic role for PD-L1 expression appears contextual and lacks consensus at this time, additional studies have explored the association between PD-L1 expression and that of other clinical or molecular features. Though select studies have affirmed the positive association between PD-L1 expression and increased lymphocyte infiltration in IBC, some suggest that PD-L1 expression is associated with an apparent anti-tumor CD8+ T-cell response [33,70]. Notably, tumors with PD-L1 upregulation had a comparative increase in the expression of several genes involved in anti-tumor cytotoxicity such as IFNγ, TNFα, and IL-2 [70]. However, despite the apparent association between PD-L1 and genes involved in a functional anti-tumor response, this study also revealed an association between PD-L1 and several genes associated with T-cell exhaustion. These include the clinically actionable immune checkpoint *CTLA4*, as well as *LAG3* and *TIM3* [70]. PD-L1 expression was also associated with several soluble immunosuppressants associated with T-cell exhaustion, namely *IDO* and *TGFβ1*. As PD-L1 expression appeared to correlate with both T-cell activation and T-cell exhaustion markers, the authors hypothesized that many TILs may be transitioning from an active to an exhausted phenotype, and the use of ICIs may offer a potential means of prolonging or reinvigorating T-cell activation in IBC [70]. In light of these studies, it is also important to note that PD-L1 status can vary significantly based on the assay used for quantification [72]. Hence, though PD-L1 is often expressed in IBC, future studies may benefit from a more standardized metric to evaluate PD-L1 status.

## 9. Clinical Trials Exploring Immunotherapy in IBC

Immunotherapy is emerging in the clinical management of breast cancer, and PD-1 or PD-L1 antagonists are beginning to show early efficacy in several difficult-to-treat breast cancer subtypes, namely those that are triple negative, PD-L1 positive, or have higher levels of TILs [73]. Interestingly, immunotherapy has long been under clinical investigation in IBC, with the earliest findings reported in the late 1970s. One of the first clinical trials exploring immunotherapy in IBC enrolled 32 patients who were administered a combination of 5-fluorouracil, doxorubicin, and cyclophosphamide (the FAC regimen) with a Bacillus Calmette-Guérin (BCG) therapeutic vaccine, followed by radiation. These patients were then compared to 32 historical controls that were treated with radiation therapy alone. The combined approach led to a modest improvement in outcomes, extending median overall survival from 18 to 24 months [74]. The authors conducted a long-term follow up of these patients, and concluded that the combination treatment was effective in prolonging the disease-free interval and survival of patient’s ≥50 years of age, with 45% surviving free of disease beyond 42 months.

This was followed by a study of 13 patients who were treated with a combination of surgery, FAC chemotherapy, and irradiated allogeneic breast cancer cells admixed with BCG, which similarly delayed disease recurrence when compared to patients treated with chemoradiotherapy or radiotherapy alone [75]. The authors followed these patients for 10 years, after which 4 of 13 (31%) were still alive. The authors therefore concluded that long-term survival is a feasible goal for patients with IBC [76]. Other studies of the era explored similar combination strategies. One such example explored neoadjuvant doxorubicin, vincristine, 5-fluorouracil, methotrexate, and melphalan combined with the I-BCG-F Pasteur immunotherapy. Chemotherapy resumed following surgery, and this approach led to favorable clinical outcomes and improved long-term survival compared to controls [77,78,79]. In the many years since these studies were conducted, BCG based immunotherapy has fallen out of favor in IBC. While there is one recent case of natural killer cell-based autologous immune enhancement therapy having a single patient with declining immune function [80], current treatment paradigms rely predominantly on chemo-, radio-, hormonal, and targeted therapy.

Given the early promise of immunotherapy in IBC and the success of ICI-based therapy in other cancers, investigators have been eager to determine whether ICIs will offer a therapeutic benefit to IBC patients. While early reports have shown promise, these trials have been limited by slow accrual, with early results often stemming from trials that included a variety of breast cancer histologies. This includes a recent Phase 2 trial exploring single-agent treatment with the anti-PD-1 antibody pembrolizumab as maintenance therapy for metastatic IBC or metastatic, non-IBC, triple-negative breast cancer (TNBC). Though only six IBC and nine TNBC patients were included in this study, the authors reported a disease control rate of 47% after 5 months, with the remaining patients experiencing a progressive disease. No objective responses were observed. When evaluating peripheral blood mononuclear cells, they also reported that patients with progressive disease had an increased proportion of CTLA4 positive CD4+ T-cells when compared to those with stable disease. Additionally, patients with high locality of CD4+ T-cells, or CD4+ T-cells that show fewer signs of functional exhaustion such as CTLA4, TIM3, and 2B4 were more likely to experience stable disease [81].

The anti-PD-1 antibody nivolumab has also been explored as a neoadjuvant treatment in IBC. In another recent Phase 2 trial, 52 patients with non-metastatic IBC were enrolled into one of two groups. The first consisted of patients with TNBC or HR-positive, HER2-negative IBC, who would receive nivolumab in combination with paclitaxel followed by doxorubicin and cyclophosphamide. The second group consisted of HER2-positive patients, who would receive nivolumab in combination with a taxane (docetaxel or paclitaxel), trastuzumab, and pertuzumab followed by doxorubicin and cyclophosphamide. After completing neoadjuvant therapy, all patients would undergo mastectomy and adjuvant radiation. While this study is underway, efficacy data have not yet been reported [82].

Another study focused exclusively on IBC, and explored the combination of the anti-PD-L1 antibody atezolizumab with the MEK inhibitor cobimetinib and eribulin in patients with recurrent or metastatic disease who had progressed on at least one line of standard chemotherapy. After an upfront biopsy, patients would receive intravenous atezolizumab and cobimetinib treatment for 4 weeks, after which they would undergo a second biopsy. At this time, patients would start standard eribulin dosing. Following four cycles of eribulin, patients receive maintenance, targeted therapy until disease progression or intolerable toxicity. As of the initial report, seven patients had been enrolled, though efficacy data are not yet available [83].

A recent study sought to evaluate the combination of nivolumab and the anti-CTLA4 antibody ipilimumab in patients with recurrent, metastatic HER2-negative IBC (NCT02892734). However, this trial was terminated in 2019 due to slow accrual, with only three patients having been treated at the conclusion of the study. Two of three patients did not continue treatment after the first 12-week cycle due to disease progression. The third patient started the second treatment cycle, but was lost to follow-up due to early termination of the study. Despite the small sample size, it is important to note that this study included two serious adverse events in the forms of acute hypoxic respiratory failure and pneumonitis with pleural effusion. Other adverse effects include leukopenia/lymphopenia, elevated aspartate aminotransferase, erythema multiforme, hypertension, hyperglycemia, hypokalemia, and hypoalbuminemia.

Several other clinical trials are ongoing or currently enrolling IBC patients, though many have yet to report results. These are mainly backboned by pembrolizumab, which is being explored as both a monotherapy and with neoadjuvant chemotherapy, hormone therapy, or various small molecule inhibitors. Two such studies include IBC patients. One of these ongoing studies is a Phase 2 trial evaluating pembrolizumab as maintenance therapy for patients with Stage IV or recurrent IBC (or TNBC) who have reached clinical response or stable disease in prior chemotherapy treatment (NCT02411656). Patients will receive single-agent pembrolizumab every 3 weeks for up to 2 years unless showing serious adverse effects or disease progression. This study was first posted in April 2015 and is still recruiting participants to reach a goal of 35 patients.

A recent Phase 2 study explored the efficacy of the AXL kinase inhibitor bemcentinib in combination with pembrolizumab in patients with previously treated TNBC (NCT03184558). This study included IBC patients and stated a goal of 56 participants, though only 29 were enrolled. At this time, results have not yet been published [84]. Two additional trials focusing solely on IBC are either ongoing or currently enrolling patients. This includes a Phase 3 trial exploring the combination of pembrolizumab with neoadjuvant chemotherapy in HER2-negative IBC (NCT03515798). Patients will receive pembrolizumab every three weeks while receiving epirubicine and cyclophosphamide, which will be administered every 2 weeks and followed by weekly paclitaxel. This study is currently recruiting with a goal of 81 participants.

Finally, a Phase 3 trial is currently evaluating pembrolizumab in combination with anti-hormone therapy in hormone receptor-positive IBC (NCT02971748). This study includes patients with Estrogen and/or Progesterone Receptor-positive tumors that did not achieve pathological complete responses after neoadjuvant chemotherapy. Patients will undergo standard radiation and hormonal therapy for their disease with concurrent intravenous pembrolizumab every 3 weeks for up to 2 years pending adverse events or disease progression. This study is currently recruiting with a goal of 37 participants. These and other ongoing efforts to explore immunotherapy in IBC are described in Table 1.

## 10. Conclusions

Despite the recent advances in other forms of breast cancer, IBC remains a significant challenge in the clinic and is often poorly responsive to conventional therapies. Though immunotherapy has revolutionized the treatment paradigm for a number of cancer histologies, such approaches have yet to show substantial therapeutic efficacy in IBC. As described, the reasons for the relative failure of immunotherapy in IBC are poorly understood. IBC is hallmarked by many of the same barriers to therapeutic efficacy that have limited immune therapy in other breast cancers; however, there are relatively few studies exploring the innate resistance of IBC to such approaches. Additionally, the immune milieu of the IBC TME is still emerging, with many results that appear highly context-specific and often contradictory.

This underscores a need to better understand not only the immune composition of IBC tumors, but also raises the question as to whether IBC should truly be considered a separate immunologic entity from other breast cancers of the same molecular subtype. One of the main limitations in answering these important questions is the rarity of IBC compared to non-IBC breast cancers. Accounting for fewer than 2% of breast cancer cases, clinical studies of immunotherapy in IBC have been plagued by issues of slow accrual. Resultantly, many of these trials have either been terminated, or conducted in combination with other breast cancer subtypes. Additionally, these non-IBC exclusive studies often report aggregate results, occasionally masking potential differences between IBC and non-IBC tumor types. Further, unlike other breast cancers, there are relatively few preclinical models of IBC. Though patient-derived xenograft models and patient-derived xenograft-derived spheroids of IBC have been reported, these models rely on immune-compromised mice and/or ex vivo culture conditions, limiting their utility for studies in tumor immunology [85,86].

Hence, this is an important and largely understudied area of breast cancer research that warrants continued exploration, particularly as IBC carries a far worse prognosis than stage-matched non-IBC breast cancer [9]. As immunotherapy continues to advance in breast cancer treatment, these and other factors should be carefully considered in the hopes of providing a much-needed therapeutic option for IBC patients who are unable to achieve pathologic complete responses using conventional methods.

## Figures and Tables

**Table 1:** Select ongoing clinical trials examining immune checkpoint inhibitors (ICIs) in inflammatory breast cancer.

Clinical Trial Number	Phase	Therapy Regimen	Notes
NCT02411656	2	Pembrolizumab	Metastatic or Recurrent HER2− IBC
NCT03742986	2	Nivolumab, Doxorubicin, Cyclophosphamide, and Paclitaxel ± Trastuzumab for HER2+	Newly Diagnosed, Non-Metastatic IBC
NCT03202316	2	Atezolizumab, Cobimetinib, and Eribulin	Previously Treated, Metastatic IBC
NCT03515798	2	Pembrolizumab, Epirubicine, Cyclophosphamide, and Paclitaxel	Non-Metastatic HER2− IBC
NCT02971748	2	Pembrolizumab and the Indicated Hormone Therapy	Non-Metastatic, HR+, HER2− IBC
NCT03184558	2	Pembrolizumab and Bemcentinib	Triple Negative IBC

Abbreviations: Inflammatory breast cancer (IBC); Immune checkpoint inhibitor (ICI); Hormone Receptor (HR); Human epidermal growth factor receptor 2 (HER2).
